# Isthmocele risk in repeated cesarean: the diagnostic and clinical role of morphometric parameters

**DOI:** 10.1007/s00404-025-08238-6

**Published:** 2025-11-08

**Authors:** Gülhan Özüm, Hakan Güraslan, Levent Deniz, Tuğba Demirtaş

**Affiliations:** 1 Clinic of Gynecology, University of Health Sciences Istanbul Bağcılar Training and Research Hospital, Istanbul, Turkey; 2 Department of Medical Biochemistry, University of Health Sciences, Istanbul Training and Research Hospital, Istanbul, Turkey

**Keywords:** Isthmocele, Cesarean section, Morphometric parameters, Residual myometrial thickness (RMT), Transvaginal ultrasonography (TVUS)

## Abstract

**Objective:**

This study aimed to evaluate the impact of repeated cesarean deliveries on isthmocele formation and to investigate the diagnostic value and clinical presentation of morphometric parameters, including niche depth, width, length, residual myometrial thickness (RMT), adjacent myometrial thickness (AMT), and the depth/AMT ratio.

**Methods:**

A cross-sectional study was conducted with 116 symptomatic and asymptomatic women aged 18–45 years who had undergone one or more cesarean sections. The presence and dimensions of the isthmocele were assessed via transvaginal ultrasonography using Delphi consensus criteria. Morphometric and obstetric data were analyzed using descriptive statistics, correlation analysis, and binary logistic regression.

**Results:**

Isthmocele was identified in 71.6% of participants, with prevalence rising from 42.9% after the first cesarean to 100% after the fourth. The isthmocele group had significantly higher gravidity, parity, and cesarean numbers (*p* < 0.001). Niche depth, length, and the depth/AMT ratio were significantly elevated, while RMT was reduced (*p* < 0.001). The number of cesareans showed a strong negative correlation with RMT (*r* = –0.499, *p* < 0.001) and a strong positive correlation with the depth/RMT ratio (*r* = 0.615, *p* < 0.001). Multivariate analysis identified having three or more cesareans as an independent predictor of isthmocele (OR = 15.6; 95% CI 3.27–74.4; *p* < 0.001). Niche length had the highest diagnostic accuracy for symptomatic isthmocele (AUC 0.700; 95% CI 0.589–0.796; cutoff 5 mm).

**Conclusion:**

Repeated cesarean deliveries significantly increase both the risk and severity of isthmocele. In women with four cesareans, isthmocele was detected in 100% of cases. A niche length of ≥ 5 mm proved to be the most reliable morphometric marker in identifying symptomatic cases.

**Implications for clinical practice:**

These findings emphasize the importance of routine transvaginal ultrasound screening post-cesarean—especially in women with multiple cesarean sections—and the incorporation of morphometric assessment (including RMT and depth/RMT ratio) into clinical decision-making.

## What does this study adds to the clinical work

**Table:** 

This study demonstrated that the risk of isthmocele increases cumulatively with repeated cesarean deleveries and that a niche length ≥ 5 mm is the most reliable indicator of symptomatic cases. These findings support the incorporation of morphometric ultrasoung assessment into post-cesarean follow-up and clinical decision-making.	

## Introduction

Isthmocele is an anechoic indentation at the site of a previous cesarean scar, communicating with the uterine cavity and reflecting myometrial discontinuity. It is regarded as an iatrogenic defect [1, 2], first described by Poidevin in 1961 [3]. In the literature, it is also described using terms such as cesarean scar defect, niche, uteroperitoneal fistula, sacculation, pouch, and diverticulum [1]. Isthmocele is often asymptomatic and usually detected incidentally on transvaginal ultrasound (TVUS) [4]. In symptomatic cases, serious obstetric complications may occur, including scar dehiscence, third-trimester uterine rupture, cesarean scar pregnancy–related hemorrhage, and placental adhesion disorders [5]. Gynecological complications, recently better defined, include postmenstrual bleeding, dysmenorrhea, dyspareunia, pelvic pain, infertility, adenomyosis, endometriosis, and abscess formation [6–8].

Although the exact risk factors remain unclear, various contributing factors have been proposed in previous studies [4]. Multiple cesarean deliveries and uterine retroflexion are regarded as key risk factors [9, 10]. Other risk factors may include advanced labor during surgery, low uterine incision, cervical dilation > 5 cm, and incomplete hysterotomy closure. Early uterine adhesions and possible genetic predisposition have also been proposed [7, 11]. Metabolic and obstetric factors, including obesity, gestational diabetes, preeclampsia, and multiparity, may also increase isthmocele risk. Surgical factors such as elective cesarean, prolonged labor > 3 h [5], short surgery duration [12], suture material, and uterine closure technique also impact isthmocele formation [2, 10].

TVUS, sonohysterography (SHG), hysterosalpingography (HSG), hysteroscopy, and magnetic resonance imaging (MRI) are established imaging modalities frequently utilized for isthmocele diagnosis [5, 6]. Among these modalities, SHG demonstrates greater sensitivity and specificity than TVUS. The prevalence of isthmocele ranges from 56 to 84% with SHG and from 24 to 70% with TVUS [13]. Despite its lower sensitivity, TVUS remains the first-line diagnostic tool due to its wide availability, simplicity, rapid application, cost-effectiveness, and non-invasive nature [14].

This study aimed to assess the impact of cesarean delivery numbers on isthmocele development and to evaluate the diagnostic and clinical relevance of morphometric parameters—including isthmocele dimensions, residual myometrial thickness (RMT), adjacent myometrial thickness (AMT), and related ratios—about symptoms.

## Materials and methods

**Fig. 1:**
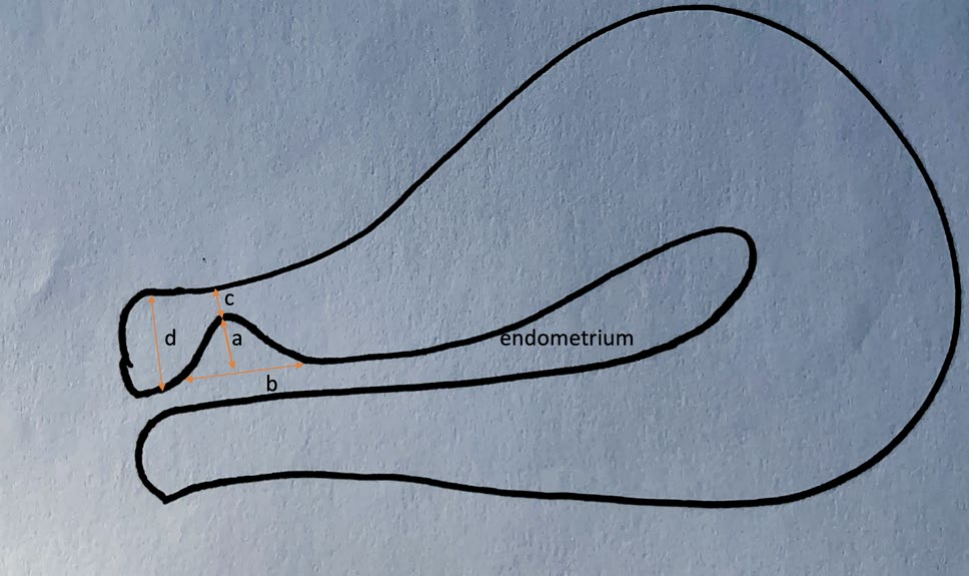
Schematic representation of isthmocele measurement based on the Delphi consensus. The drawing is inspired by Antila-Långsjö et al. [7]. In the sagittal plane—**a** depth: vertical distance between the base and the apex of the defect; **b** length: distance between the proximal edge of the scar (near the cervix) and the distal edge (near the uterine fundus); **c** residual myometrial thickness: shortest distance between the endometrium and the uterine serosa; **d** adjacent myometrial thickness

**Fig. 2:**
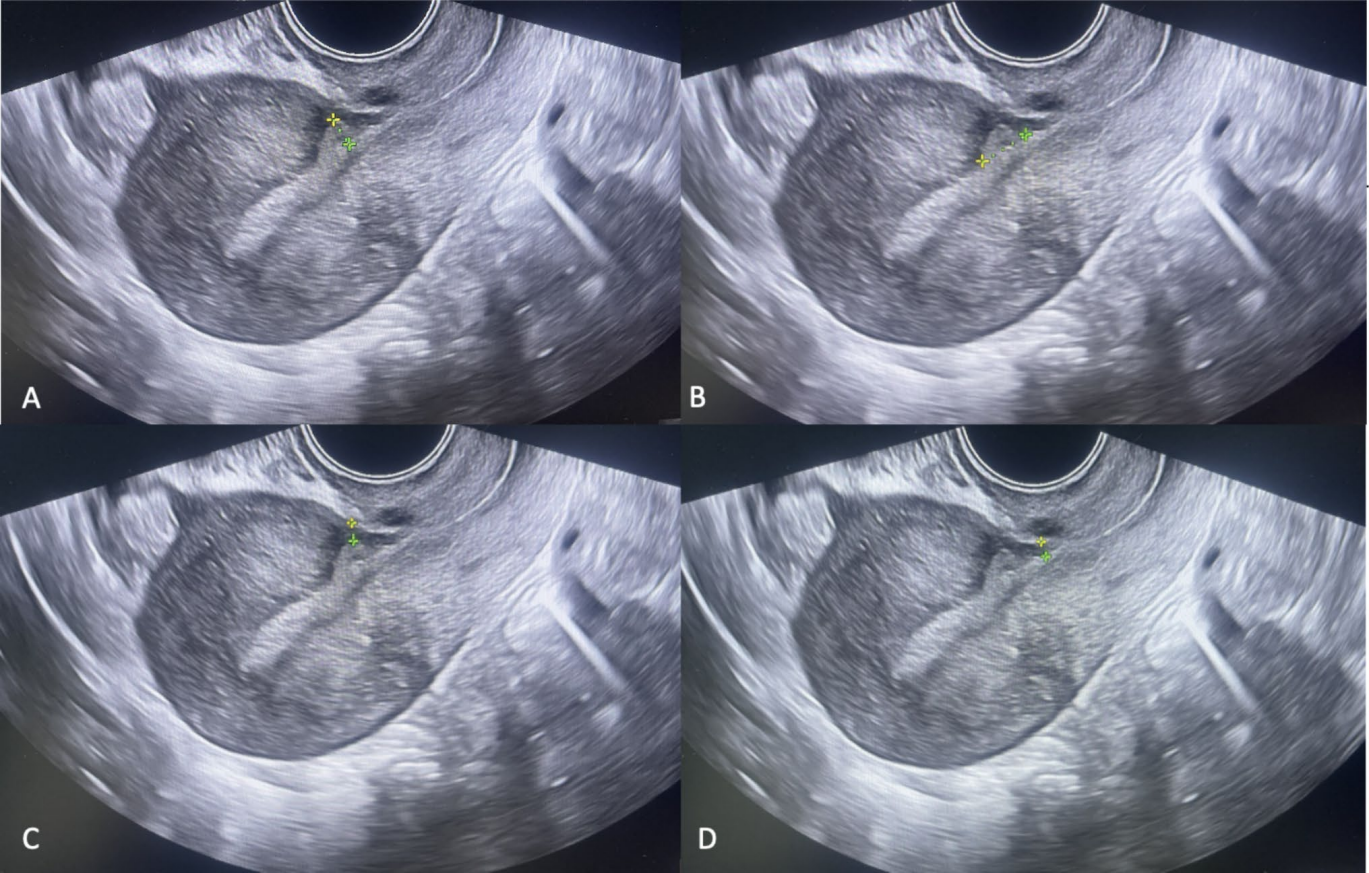
Ultrasonographic representation of isthmocele measurements in the sagittal plane. **A** Depth; **B** length; **C** residual myometrial thickness (RMT); **D** adjacent myometrial thickness (AMT)

The study was conducted between March and December 2024, with ethical approval from the Institutional Review Board. Written informed consent was obtained from all participants. The study included women aged 18–45 years with a history of ≥ 1 cesarean delivery who underwent TVUS for gynecological indications. Isthmocele was defined as an anechoic or hypoechoic triangular defect located at the site of the previous cesarean section scar, with a minimum depth of 2 mm and a clear communication with the endometrial cavity. Niche measurements followed the standardized criteria outlined by the Delphi consensus [7, 15] (Fig. 1). The measurements were performed by a gynecologist experienced in the field, with the patient in the dorsal lithotomy position and an empty bladder. The residual myometrial thickness (RMT) was measured as the shortest distance between the endometrial interface and the serosa at the scar site. Adjacent myometrial thickness (AMT), niche depth (vertical distance from base to apex), niche length (from proximal cervical to distal fundal margin in the sagittal plane), and width (widest point in the transverse plane) were also recorded (Fig. 2). If multiple niches were present, only the largest was included for analysis. Symptomatic status was based on self-reported complaints. Women reporting irregular cycles, postmenstrual spotting, pelvic pain, or dysmenorrhea were classified as symptomatic. Women were included if ≥ 6 months had passed since their last cesarean and they had no history of anterior uterine wall surgery. No upper time limit was set after the last cesarean. Ultrasound exams were conducted by a single expert using the SonoScape S40 color Doppler system (SonoScape Medical Corp., Shenzhen, China) with a 5–9 MHz transducer for TVUS. Data on body mass index (BMI), cesarean indication, obstetric history, cesarean delivery numbers, and patient-reported symptoms were collected via medical records and structured interviews. Information regarding the indications for cesarean section was collected from the patients. These indications included breech presentation, fetal distress, non-progressive labor, placental abruption, cephalopelvic disproportion (CPD), fetal macrosomia, twin pregnancy, and other causes. TVUS was performed once in the early follicular phase when intrauterine fluid was present. Uterine position was also documented. Normal vaginal delivery (NVD) is defined as a vaginal birth that occurs without the use of any delivery instruments (e.g., vacuum or forceps), regardless of whether labor was induced with pharmacological agents or not. Emergency cesarean was defined as surgery for urgent indications (e.g., fetal distress, prolonged labor), while elective cesarean referred to planned procedures (e.g., macrosomia, multiple pregnancy). Since this study aims to evaluate only the risk factors and maternal outcomes associated with isthmocele, neonatal outcomes were excluded from the scope of analysis.

### Sample size

Sample size calculation to compare two independent groups was performed using G*Power software version 3.1.9.7 (Heinrich-Heine-Universität Düsseldorf, Düsseldorf, Germany). The effect size was set as Cohen’s *d* = 0.75, based on the study by Fakhr et al. [16]. Assuming a significance level (*α*) of 0.05 and power (1-β) of 0.80, the minimum total sample size required was calculated as 58.

### Statistical analysis

Data normality was evaluated by the Shapiro–Wilk test. Normally distributed data are presented as mean ± standard deviation, while non-normal data are shown as median (interquartile range, 25–75th percentiles). Categorical variables were analyzed using Chi-square or Fisher’s exact test. When the assumption of the Chi-square test was violated (i.e., > 20% of the cells had an expected count < 5), Fisher’s exact test was utilized. For two-group comparisons, Student’s *t *test was used for parametric data, and Mann–Whitney U test was used for non-parametric data. For comparisons among four groups, one-way ANOVA was used for parametric data and the Kruskal–Wallis test was used for non-parametric data. Post hoc pairwise analyses employed Bonferroni or Tamhane’s T2 tests for one-way ANOVA. For pairwise comparisons between the four groups, Bonferroni correction was applied, setting the statistical significance threshold at *p* < 0.008. Spearman’s correlation was used to assess variable relationships. Receiver operating characteristic (ROC) curve analysis determined the optimal diagnostic cutoffs for morphometric parameters in symptomatic isthmocele cases. Binary logistic regression identified the independent isthmocele risk factors. A multicollinearity test was conducted on the independent variables using the variance inflation factor (VIF), and only variables with a VIF value below 3 were included in the multivariate analysis, which was performed using Jamovi software (version 2.3.28). Analyses were performed using SPSS v26 (IBM Corp., Armonk, NY, USA). Significance was set at *p* < 0.05.

## Results

A total of 116 women with a cesarean history were included; 83 (71.6%) in the isthmocele group and 33 (28.4%) in the control group (Table 1). The cesarean section numbers were: one (36.2%), two (25.0%), three (21.6%), and four (17.2%). The prevalence of isthmocele increased with the number of cesarean deliveries: 42.9% after one, 75.9% after two, 92.0% after three, and 100% after four (Table 2). Although not statistically significant, emergency cesarean sections were more frequent (54%) than elective procedures (46%) in the isthmocele group. Repeat cesarean was the leading indication for elective surgery (78%), while fetal distress was the most common cause of emergency cesarean (25%). Gravida, parity, number of cesarean deliveries, and history of repeat cesarean were significantly higher, while time since surgery was shorter in the isthmocele group compared to the non-isthmocele group (*p* < 0.05). No significant differences were found between groups regarding age, BMI, gestational diabetes, cesarean type, cervical dilation, miscarriage or curettage history, uterine position, or hematological markers (Table 1).

**Table 1:** Comparison of demographic, clinical, and laboratory characteristics between the groups

Variables	Isthmocele (−) group (*n* = 33)	Isthmocele ( +) group (*n* = 83)	p*
Age (years)	33 (28–41)	35 (30–40)	0.558
BMI (kg/m^2^)	26.9 ± 4.22	27.0 ± 4.58	0.291
Symptoms, *n* (%)			
Vaginal spotting	0 (0.00)	5 (6.02)	0.319
Bleeding	3 (9.09)	8 (9.64)	0.617
Pelvic pain	2 (6.06)	16 (19.3)	0.136
Irregular menstruation	7 (21.2)	15 (18.1)	0.899
Dysmenorrhea	0 (0.00)	1 (1.20)	0.716
Gravid (*n*)	2.00 (1.00–4.00)	3.00 (2.00–4.00)	0.007
Parity (*n*)	2.00 (1.00–2.25)	3.00 (2.00–4.00)	< 0.001
GDM, *n* (%)	0 (0.00)	5 (7.60)	0.317
Time since surgery (years)	4.50 (2.50–9.00)	3.00 (1.00–6.00)	0.022
Number of CS (*n*)	1.00 (1.00–2.00)	3.00 (2.00–3.00)	< 0.001
Type of CS, *n* (%)			0.347
Elective CS	19 (57.6)	38 (45.8)	
Emergency CS	14 (42.4)	45 (54.2)	
Indication for CS, *n* (%)			
Breech presentation	6 (18.2)	11 (13.3)	0.563
Fetal distress	5 (15.2)	21 (25.3)	0.349
Non-progressive labor	9 (27.3)	20 (24.1)	0.905
Placental abruption	0 (0.00)	4 (4.80)	0.577
CPD	3 (9.10)	2 (2.40)	0.138
Fetal macrosomia	2 (6.10)	2 (2.40)	0.320
Twin	2 (6.10)	2 (2.40)	0.320
Others**	3 (9.10)	6 (7.20)	0.712
Elective CS	3 (9.10)	15 (18.1)	0.357
Repetition CS	9 (27.3)	65 (78.3)	< 0.001
Cervical dilatation (cm)	1.00 (0.00–3.00)	0.00 (0.00–2.75)	0.645
NVD (*n*)	0.00 (0.00–1.00)	0.00 (0.00–0.00)	0.005
Miscarriages/curettage (*n*)	0.00 (0.00–1.00)	0.00 (0.00–1.00)	0.867
Uterine position (*n*, %)			0.490
Anteversion	32 (97.0)	82 (98.8)	
Retroversion	1 (3.00)	1 (1.20)	
Niche depth (mm)	1.40 (1.20–1.80)	3.50 (2.90–4.30)	< 0.001
Niche length (mm)	2.80 (2.38–3.30)	4.20 (3.33–5.60)	< 0.001
Niche width (mm)	4.10 (3.68–4.20)	4.30 (3.53–5.30)	0.079
RMT (mm)	4.70 (4.20–6.93)	3.50 (2.63–4.90)	< 0.001
AMT (mm)	6.70 (5.88–8.25)	7.50 (6.00–8.70)	0.426
Depth/RMT	0.28 (0.21–0.34)	1.02 (0.70–1.38)	< 0.001
Depth/AMT	0.20 (0.16–0.25)	0.51 (0.41–0.58)	< 0.001
RMT/AMT	0.77 (0.70–0.84)	0.50 (0.42–0.60)	< 0.001
Hemoglobin (g/dL)	11.9 (11.0–13.0)	11.4 (10.1–12.5)	0.137
Hematocrit (%)	35.9 ± 4.57	34.5 ± 4.30	0.181
Leukocyte count (10^9^/L)	8.87 ± 2.88	10.0 ± 3.05	0.120

*BMI* body mass index, *GDM* gestational diabetes mellitus, *CS* cesarean section, *CPD* cephalopelvic disproportion, *NVD* normal vaginal delivery, *RMT* residual myometrial thickness, *AMT* adjacent myometrial thickness

^*^P < 0.05: statistically significant

^**^Others: preeclampsia, umbilical cord entanglement, intrauterine growth restriction, in vitro fertilization pregnancy

**Table 2:** Comparison of demographic, clinical, and laboratory characteristics among groups stratified by the number of CS

Variables	1 CS (*n* = 42)	2 CSs (*n* = 29)	3 CSs (*n* = 25)	4 CSs (*n* = 20)	*p* value*
Age (years)	30 (27–39)	37 (30–41)	38 (32–42)	35 (33–40)	0.062
BMI (kg/m^2^)	25.5 (22.8–28.3)	26.2 (23.9–29.7)	27.5 (24.4–31.1)	28.9 (24.3–29.7)	0.085
Gravid (*n*)	1.00 (1.00–3.00)	2.00 (2.00–3.00)	4.00 (3.00–4.00)^a2^	4.00 (4.00–5.00)^a2,b2^	< 0.001
Symptoms, *n* (%)	16 (38.1)	7 (24.1)	19 (76.0)^a1,b2^	15 (75.0) ^b1^	< 0.001
Parity (*n*)	1.00 (1.00–2.00)	2.00 (2.00–2.00)^a1^	3.00 (3.00–3.00)^a2,b1^	4.00 (4.00–4.00)^a2,b2^	< 0.001
GDM, *n* (%)	1 (3.20)	2 (8.30)	2 (11.1)	0 (0.00)	0.404
Time since surgery (years)	4.50 (2.00–8.00)	4.00 (1.00–6.00)	3.50 (1.88–7.00)	1.50 (1.00–4.00)^a1^	0.035
Type of CS, *n* (%)					0.007
Elective CS	29 (69.0)	10 (34.5)^a1^	12 (48.0)	6 (30.0)^a1^	
Emergency CS	13 (31.0)	19 (65.5)	13 (52.0)	14 (70.0)	
Indication for CS, *n* (%)					
Breech presentation	11 (26.2)	1 (3.40)	2 (8.00)	3 (15.0)	0.041
Fetal distress	7 (16.7)	9 (31.0)	6 (24.0)	4 (20.0)	0.544
Non-progressive labor	6 (14.3)	8 (27.6)	7 (28.0)	8 (40.0)	0.158
Placental abruption	0 (0.00)	2 (6.90)	0 (0.00)	2 (10.0)	0.066
CPD	3 (7.10)	1 (3.40)	1 (4.00)	0 (0.00)	0.815
Fetal macrosomia	2 (4.80)	0 (0.00)	1 (4.00)	1 (5.00)	0.758
Twin	3 (7.10)	1 (3.40)	0 (0.00)	0 (0.00)	0.491
Others**	1 (2.40)	2 (6.90)	5 (20.0)	1 (5.00)	0.076
Elective CS	9 (21.4)	5 (17.2)	3 (12.0)	1 (5.00)	0.403
Cervical dilatation (cm)	0.00 (0.00–2.00)	0.00 (0.00–2.00)	0.00 (0.00–3.25)	0.00 (0.00–3.00)	0.837
NVD (*n*)	0.00 (0.00–1.00)	0.00 (0.00–0.00)	0.00 (0.00–0.00)^a1^	0.00 (0.00–0.00)^a1^	0.008
Miscarriages/curettage (*n*)	0.00 (0.00–1.00)	0.00 (0.00–1.00)	1.00 (0.00–1.00)	0.00 (0.00–1.00)	0.235
Uterine position (*n*, %)					0.660
Anteversion	40 (95.2)	29 (100)	25 (100)	20 (100)	
Retroversion	2 (4.80)	0 (0.00)	0 (0.00)	0 (0.00)	
Isthmocele, (*n*, %)	18 (42.9)	22 (75.9)^a1^	23 (92.0)^a2^	20 (100)^a2^	< 0.001
Niche depth (mm)	1.80 (1.30–3.00)	2.50 (2.05–3.90)	3.60 (3.05–4.30)^a2^	4.30 (2.95–5.10)^a2,b1^	< 0.001
Niche length (mm)	3.10 (2.50–3.80)	3.50 (2.78–3.93)	4.50 (3.20–6.03)^a1^	5.50 (4.50–7.35)^a2,b2^	< 0.001
Niche width (mm)	4.10 (3.50–4.80)	3.90 (3.40–4.53)	4.20 (3.90–5.23)	4.75 (4.00–7.65)	0.072
RMT (mm)	5.30 ± 1.89	4.20 ± 1.53	3.65 ± 0.78^a2^	3.07 ± 1.28^a2^	< 0.001
AMT (mm)	7.45 (6.10–9.00)	6.90 (5.68–8.73)	7.20 (6.15–8.33)	7.50 (5.90–8.35)	0.830
Depth/RMT	0.41 (0.23–0.57)	0.72 (0.46–1.08)	1.05 (0.83–1.26)^a2^	1.50 (1.05–1.94)^a2,b2^	< 0.001
Depth/AMT	0.30 (0.18–0.36)	0.42 (0.34–0.52)	0.51 (0.45–0.56)^a2^	0.60 (0.51–0.66)^a2,b1^	< 0.001
RMT/AMT	0.70 (0.58–0.82)	0.58 (0.48–0.67)	0.52 (0.46–0.55)^a2^	0.40 (0.34–0.49)^a2,b1^	< 0.001
Hemoglobin (g/dL)	11.6 ± 1.62	11.5 ± 1.89	11.2 ± 1.48	11.2 ± 1.26	0.768
Hematocrit (%)	35.6 ± 4.92	35.2 ± 4.65	34.2 ± 4.16	34.2 ± 3.52	0.618
Leukocyte count (10^9^/L)	9.18 ± 3.15	10.1 ± 2.99	9.95 ± 2.75	9.75 ± 3.27	0.690

^BMI body mass index, *GDM* gestational diabetes mellitus, *CS* cesarean section, *CPD* cephalopelvic disproportion, *NVD* normal vaginal delivery, *RMT* residual myometrial thickness, *AMT* adjacent myometrial thickness^

^*P<0.05: statistically significant. Bonferroni correction was applied for pairwise comparisons of the four groups, setting significance at *p*<0.008. a1: *p*<0.008, a2: *p*<0.001, a: comparison with the 1 CS group, b1: *p*<0.008, b2: *p*<0.001, b: comparison with the 2 CSs group^

^**Others: preeclampsia, umbilical cord entanglement, intrauterine growth restriction, in vitro fertilization pregnancy^

In the isthmocele group, median niche length [4.20 mm (IQR 3.33–5.60)] and depth-to-AMT ratio [0.51 (IQR 0.41–0.58)] were increased, while RMT was decreased [3.50 mm (IQR 2.63–4.90)]. No significant differences in niche width or AMT were observed between groups (Table 1). Niche size parameters increased significantly with the number of cesarean sections. Median niche depth increased progressively with cesarean number: 1.80 mm (IQR 1.30–3.00) after one, 2.50 mm (IQR 2.05–3.90) after two, 3.60 mm (IQR 3.05–4.30) after three, and 4.30 mm (IQR 2.95–5.10) after four sections. Niche depth, length, and depth-to-AMT ratio were significantly higher, and RMT was significantly lower in women with three or four prior cesarean deliveries compared to those with one or two (*p* < 0.05). No statistically significant differences were found in isthmocele-related measurements between women with one and two previous cesarean sections (Table 2). The number of cesarean sections correlated negatively with RMT (*r* = –0.499, p < 0.001) and positively with niche depth (*r* = 0.540), length (*r* = 0.519), width (*r* = 0.198), depth-to-AMT ratio (*r* = 0.608), and depth-to-RMT ratio (*r* = 0.615). The index showing the strongest correlation with the number of cesarean sections was the depth-to-RMT ratio (*r* = 0.615, *p* < 0.001) (Table 3).

**Table 3:** Significant correlations between CS number and the other variables in all groups

Parameter	CS number	
	*r*	*p*
Age (years)	0.211	0.023
BMI (kg/m^2^)	0.237	0.010
Gravid (*n*)	0.643	< 0.001
Parity (*n*)	0.798	< 0.001
NVD (*n*)	− 0.319	< 0.001
Niche depth (mm)	0.540	< 0.001
Niche length (mm)	0.519	< 0.001
Niche width (mm)	0.198	0.034
RMT (mm)	-0.499	< 0.001
Depth/RMT	0.615	< 0.001
Depth/AMT	0.608	< 0.001
RMT/AMT	− 0.580	< 0.001

*CS* cesarean section, *BMI* body mass index, *NVD* normal vaginal delivery, *RMT* residual myometrial thickness, *AMT* adjacent myometrial thickness

*r*: Spearman’s rank correlation coefficient

**Fig. 3:**
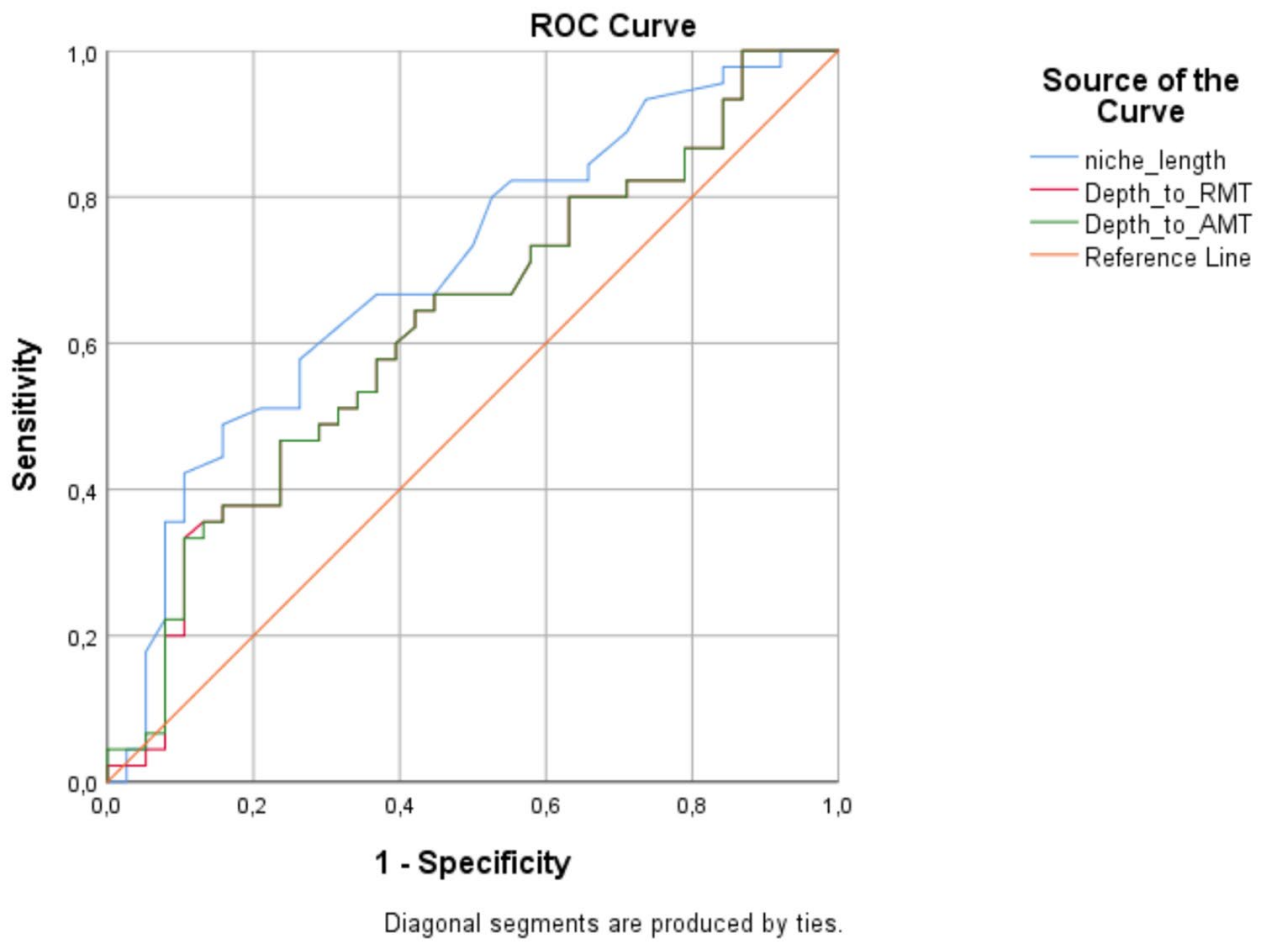
Receiver operating characteristic analysis for identifying symptomatic isthmocele patients

Niche morphology differed significantly between symptomatic and asymptomatic groups, with symptomatic women exhibiting higher median niche length (5.00 vs. 3.75 mm), depth-to-AMT ratio (0.52 vs. 0.47), and depth-to-RMT ratio (1.10 vs. 0.90) (Table 4). No significant differences were found in niche depth, width, AMT, RMT, or RMT-to-AMT ratio. Although no significant difference in RMT was observed between groups (*p* = 0.276), the mean RMT was lower in the symptomatic group (3.63 ± 1.35) compared to the asymptomatic group (3.96 ± 1.41) (Table 4). Among the predictive parameters, niche length demonstrated the highest diagnostic accuracy for symptomatic isthmocele (AUC = 0.700; 95% CI, 0.589–0.796; cutoff = 5 mm). Depth-to-AMT (AUC = 0.628; cutoff = 0.55) and depth-to-RMT ratios (AUC = 0.626; cutoff = 1.22) also showed moderate discriminative performance (Fig. 3).

**Table 4:** Comparison of demographic, clinical, and laboratory characteristics of patients with isthmocele according to the presence of symptoms

Variables	Asymptomatic group (*n* = 38)	Symptomatic group (*n* = 45)	p*
Age (years)	32 (28–39)	37 (33–41)	0.029
BMI (kg/m^2^)	26.6 ± 4.67	27.2 ± 4.53	0.561
Gravid (*n*)	3.00 (2.00–4.00)	4.00 (2.75–4.00)	0.018
Parity (*n*)	2.00 (2.00–3.00)	3.00 (2.00–4.00)	0.007
GDM, *n* (%)	1 (3.20)	4 (11.4)	0.360
Time since surgery (years)	3.25 (1.00–6.00)	2.50 (1.38–6.25)	0.904
Number of CS (*n*)	2.00 (1.00–3.00)	3.00 (2.00–4.00)	0.003
Type of CS, *n* (%)			0.024
Elective CS	23 (60.5)	15 (33.3)	
Emergency CS	15 (39.5)	30 (66.7)	
Indication for CS, *n* (%)			0.109
Breech presentation	8 (21.1)	3 (6.70)	
Fetal distress	7 (18.4)	14 (31.1)	0.284
Non-progressive labor	6 (15.8)	14 (31.1)	0.171
Placental abruption	2 (5.30)	2 (4.40)	0.625
CPD	1 (2.60)	1 (2.20)	0.709
Fetal macrosomia	1 (2.60)	1 (2.20)	0.709
Twin	1 (2.60)	1 (2.20)	0.709
Others**	5 (13.2)	1 (2.20)	0.089
Elective CS	7 (18.4)	8 (17.8)	0.940
Repetition CS	28 (73.7)	37 (82.2)	0.501
Cervical dilatation (cm)	0.00 (0.00–2.00)	0.00 (0.00–3.00)	0.494
NVD (*n*)	0.00 (0.00–0.00)	0.00 (0.00–0.00)	0.088
Miscarriages/curettage (*n*)	0.00 (0.00–1.00)	0.00 (0.00–1.00)	0.690
Uterine position (*n*, %)			0.542
Anteversion	38 (100)	44 (97.8)	
Retroversion	0 (0.00)	1 (2.20)	
Niche depth (mm)	3.35 (2.50–4.20)	3.60 (2.98–4.40)	0.170
Niche length (mm)	3.75 (2.80–4.70)	5.00 (3.70–6.48)	0.002
Niche width (mm)	4.05 (3.50–5.30)	4.50 (3.83–5.30)	0.437
RMT (mm)	3.96 ± 1.41	3.63 ± 1.35	0.276
AMT (mm)	7.50 (6.00–9.10)	7.70 (5.98–8.63)	0.873
Depth/RMT	0.90 (0.64–1.22)	1.10 (0.80–1.53)	0.049
Depth/AMT	0.47 (0.39–0.55)	0.52 (0.44–0.61)	0.045
RMT/AMT	0.52 ± 0.12	0.49 ± 0.13	0.219
Hemoglobin (g/dL)	11.1 ± 1.62	11.4 ± 1.54	0.457
Hematocrit (%)	34.2 ± 4.33	34.8 ± 4.31	0.530
Leukocyte count (10^9^/L)	10.3 ± 2.64	9.76 ± 3.38	0.492

*BMI* body mass index, *GDM* gestational diabetes mellitus, *CS* cesarean section, *CPD* cephalopelvic disproportion, *NVD* normal vaginal delivery, RMT residual myometrial thickness, *AMT* adjacent myometrial thickness

^*^P < 0.05: statistically significant

^**^Others: preeclampsia, umbilical cord entanglement, intrauterine growth restriction, in vitro fertilization pregnancy

Among 83 women with isthmocele, 45 (54.2%) were symptomatic and 38 (45.8%) were asymptomatic. The most frequent symptoms included pelvic pain (19%), menstrual irregularity (18%), abnormal vaginal bleeding (10%), spotting (6%), and dysmenorrhea (1%). Symptomatic women had significantly higher age, gravidity, parity, cesarean delivery numbers, and emergency cesarean rates. No significant differences were observed between groups in BMI, gestational diabetes, time since surgery, cervical dilation, miscarriage or curettage history, uterine position, or hematological parameters (Table 4). The incidence of symptoms was significantly higher in women with one, three, or four cesarean sections compared to those with two cesarean sections (Table 2).

Univariate logistic regression revealed significant associations between isthmocele and gravidity (OR 1.51; 95% CI 1.12–2.05), parity (OR 2.14; 95% CI 1.40–3.27), number of cesarean sections (OR 16.7; 95% CI 3.74–74.2), repeat cesarean (OR 9.63; 95% CI 3.81–24.3), and time since surgery (OR 0.90; 95% CI 0.82–0.99). Multivariate analysis identified three to four cesarean sections (OR 15.6; 95% CI 3.27–74.4; *p* = 0.001) as an independent predictor of isthmocele, independent of age, BMI, time after surgery, type of CS (Table 5).

**Table 5:** Binary logistic regression analysis for patients with isthmocele

Variables	Univariate (unadjusted)		Variables	Multivariate (adjusted)*	
	OR (95% CI)	*p *value		OR (95% CI)	*p *value
Gravid (*n*)	1.513 (1.116–2.050)	0.008	Number of CS (*n* = 3–4)	15.60 (3.270–74.42)	0.001
Parity (*n*)	2.141 (1.403–3.266)	< 0.001			
Number of CS (*n* = 3–4)	16.66 (3.743–74.18)	< 0.001			
Repetition CS	9.630 (3.811–24.33)	< 0.001			
Time since surgery (years)	0.899 (0.816–0.990)	0.030			

*OR* odds ratio, *CS* cesarean section

^*^Adjusted for age, body mass index, time since surgery, type of CS (emergency CS/elective CS). Nagelkerke *R*^2^: 0.314, Hosmer and Lemeshow *p*-value: 0.747, Omnibus test *p* value: < 0.001

## Discussion

### Risk factors and prevalence

This study assessed the association between isthmocele formation and obstetric factors, alongside correlations between morphometric measurements and clinical symptoms. Gravidity, parity, cesarean number, and repeated cesarean rate were significantly higher in the isthmocele group. These findings suggest that multiple cesarean deliveries may lead to morphological disruption at the uterine scar, particularly by reducing RMT. Consistently, in our study, the isthmocele group had significantly lower RMT and greater niche length and depth-to-AMT ratio, while niche width and AMT did not differ significantly between groups. As the number of cesarean sections increased, a significant increase was observed in niche depth, length, width, and depth-based ratios, while RMT showed a decrease. Correlation analysis showed a negative association between cesarean numbers and RMT, and a positive association with niche dimension and depth-to-AMT ratio. Multivariate analysis supported repeat cesarean as an independent risk factor for isthmocele development.

The isthmocele prevalence in our study (71.6%) aligns with previous reports: 24–70% by TVUSG and 56–84% by SHG [17]. The wide range in prevalence rates may be attributed to variations in study populations, diagnostic criteria, and imaging modalities [9]. The high rate in our study may be attributed to standardized diagnostic criteria (Delphi consensus), high-resolution ultrasonography, increased clinical awareness, and the inclusion of both symptomatic and asymptomatic patients. Subgroup analysis showed isthmocele prevalence of 43% after one cesarean, rising to 76%, 92.0%, and 100% after two, three, and four cesareans, respectively. Prevalence increased with the number of cesarean deliveries. This finding aligns with previous studies, which reported a prevalence of approximately 60% after one cesarean section and nearly 100% in women with three or more cesarean deliveries [2, 16]. Similarly, another study reported isthmocele prevalence rates of 63%, 76%, and 88% in women who had undergone one, two, and three cesarean sections, respectively [7]. These findings indicate that isthmocele risk increases with the number of cesarean sections, as repeated deliveries may cause structural damage to the uterine scar.

### Morphological parameters and clinical presentation

Several classification systems based on isthmocele size, symptomatology, and morphology have been proposed; however, none are universally accepted as standard [10]. In size-based classifications, a large isthmocele is defined as involving > 50–80% of myometrial thickness, though thresholds vary by diagnostic modality. An RMT of ≤ 2.2 mm by TVUSG and ≤ 2.5 mm by SHG is generally accepted as the defining criterion [11, 18]. Morphologically, isthmoceles can be classified into triangular, semi-circular, rectangular, teardrop, or inclusion cyst-like shapes [10]. Clinically, isthmocele is classified as asymptomatic when incidentally detected or symptomatic when associated with abnormal bleeding, infertility, or pelvic pain [19]. In our study, RMT was significantly lower in the isthmocele group compared to the non-isthmocele group (median 3.50 mm vs. 4.70 mm). This aligns with Vidushi et al., who suggest that RMT decreases as the size of the scar defect increases [1]. Previous studies have highlighted that increased isthmocele size is associated with major obstetric complications, including uterine scar dehiscence and uterine rupture during pregnancy [20]. Despite many isthmoceles being asymptomatic, certain morphometric parameters may be predictive of symptom development. In our study, larger isthmocele dimensions—especially increased niche length, depth-to-AMT, and depth-to-RMT ratio—were more common in symptomatic patients. However, niche depth, width, RMT, and AMT did not differ significantly between groups. Consequently, clinical heterogeneity persists, and defining the threshold at which an isthmocele becomes symptomatic remains a challenge.

Approximately one-third of isthmocele cases were asymptomatic; common symptoms included intermenstrual spotting (38.5%) and pelvic pain (30.5%) [21]. Pelvic pain and spotting were the most common symptoms in our study, which is consistent with the existing literature. Symptom development may be linked to menstrual blood retention, fibrotic-induced reduced contractions, and local inflammation [4, 19]. A meta-analysis reported unclear associations between isthmocele morphology, symptoms, and treatment; however, larger defects may increase complication risk [12]. In our study, symptoms were more frequent in patients with one, three, or four cesareans, while those with two had fewer symptoms, suggesting a non-linear association. Some studies link isthmocele morphology and symptoms; larger defects are associated with increased spotting, dysmenorrhea, and pelvic pain [22, 23]. Wang et al.’s study found isthmocele width linked to symptoms, while defect depth and RMT were not [22]. In our study, niche length was the key parameter distinguishing symptomatic cases, with a 5 mm cutoff. This contrasts with width and volume, which are more often emphasized in the literature. Niche length may influence symptoms via lesion surface area and inflammation, rather than fluid accumulation or drainage issues. The relationship between symptoms and isthmocele morphometry remains unclear, with studies reporting varied associations. Surface area, rather than morphometric features, may be the key parameter in symptomatic isthmoceles; prospective studies are needed in this area.

### Cesarean sections and ısthmocele development

In our study, the number of cesarean deliveries was strongly associated with isthmocele development and identified as an independent risk factor. Isthmoceles were observed in 43% after the first and 100% after the fourth cesarean section. This increase may result from repeated incisions at the same site, causing fibrosis, myometrial thinning, and poor healing due to reduced vascularization [7, 17, 24]. Prospective studies have shown that repeated cesareans increase isthmocele risk [7, 17]. The literature indicates that repeated cesareans not only increase isthmocele risk but also enlarge its dimensions [25, 26]. Scar defect assessment with transvaginal ultrasound-based calculators shows isthmocele size and development increase with the number of cesarean sections [14]. Consistent with previous studies, our results showed a significant increase in isthmocele prevalence and specific morphometric parameters like niche depth, length, and depth-to-AMT ratio. In a large study of 4250 cases, cesarean sections significantly increased isthmocele size but had no significant effect on RMT [22]. Chen’s study indicated that an interval exceeding 5 years between cesarean sections impairs wound healing [24]. In contrast, our study attributes the shorter interval in the isthmocele group to multiparity and does not consider it as a direct factor in isthmocele formation.

Studies report conflicting findings on the association between cesarean type (elective vs. emergency) and isthmocele development. This difference may stem from incision level variations: elective cesareans are typically performed at the uterovesical fold near the internal cervical os, while emergency cesareans may involve lower incisions [11, 27]. Elective cesarean section may increase the risk of isthmocele due to being performed before cervical dilation and full development of the lower uterine segment. A closed cervix can hinder intrauterine drainage, causing blood accumulation at the incision site, which creates mechanical pressure, impairs healing, and promotes isthmocele formation [25]. Conversely, other studies found no statistically significant association [4, 5, 7]. These discrepancies may result from non-standardized definitions of elective and emergency cesarean sections across studies. For instance, a cesarean planned as elective for breech presentation may be reclassified as an emergency if labor begins and dilation occurs. This reclassification poses methodological challenges. In our study, isthmocele development was not associated with elective cesarean. This finding may reflect population differences, as the elective group included patients with cervical dilation, whereas the emergency group did not.

### Anatomical and physiological factors

Uterine position may influence isthmocele development [28, 29]. Approximately one-third of women have a retroverted or intermediate uterine position [10]. In retroverted uteri, increased mechanical stress on the scar line may reduce tissue perfusion and oxygenation, impairing wound healing and increasing the risk of isthmocele formation [22, 25, 26]. A review reported a higher isthmocele incidence in women with a retroverted uterus [18]. In a study of 120 symptomatic patients, isthmocele occurred nearly twice as often in women with a retroverted uterus [29]. Conversely, a prospective study of 52 patients found no significant association, likely due to the small sample size [10]. Similarly, another study found no significant association between retroverted uterus and isthmocele, suggesting that factors such as surgical technique, suture material, and incision site may be more influential [4]. In our study, no significant association was found between isthmocele and retroverted uterus, possibly due to the limited number of retroverted cases. Whether a retroverted uterus is a cause or consequence of isthmocele remains unclear, highlighting the need for more robust studies in populations with high retroversion prevalence.

The link between isthmocele and cervical dilation remains inconclusive**.** When cervical dilation exceeds 5 cm, the myometrium thins and vascularity decreases [1, 18]. This, along with increased blood loss and risk of infection, may impair wound healing and facilitate isthmocele development [19, 24]. A review suggested that cervical mucus infiltrates between the myometrial layers, disrupting wound healing, and this mechanism may increase the risk of isthmocele in patients with cervical dilation [13]. A prospective study reported increased isthmocele incidence in women with cervical dilation > 3 cm, reaching 50% for large isthmoceles at > 8 cm. This may result from the cervix becoming part of the lower uterine segment during labor, impairing scar healing [24]. However, a prospective study found no significant association between cervical dilation and isthmocele, with a 6.7% prevalence in cases > 8 cm dilation, attributed to the small number of advanced labor cases [17]. Another study reported increased isthmocele risk with cervical dilation < 7 cm, but no added risk beyond 8 cm, likely due to higher incision placement at full dilation [4]. In our study, no significant relationship was observed, possibly due to the few dilated cases.

### Other factors

Obesity and gestational diabetes are potential modifiable risk factors for isthmocele, possibly due to chronic inflammation, infection risk, and immune/vascular changes. In addition, hyperglycemia and insulin resistance can impair wound healing [7, 17]. A study reported that each 1-unit BMI increase raised the isthmocele risk by 6%, and gestational diabetes significantly increased the risk [7]. On the other hand, another study found that obesity was not a significant risk factor [24]. Our findings are consistent with prior studies. This finding suggests that obesity-related inflammation and healing processes do not affect isthmocele development uniformly in all cases.

### Limitations and strengths

Main limitations include the single-center, cross-sectional design and small subgroup sizes (e.g., retroverted uterus, advanced dilation, gestational diabetes), which limited assessment of their association with isthmocele. The predominant use of single-layer locked continuous sutures (polyglactin/Vicryl) limited comparative analysis. In addition, insufficient data on surgical techniques restricted the evaluation of scar healing factors. Although cutoff values were identified for symptomatic cases, our study design limited their assessment regarding complication risk and treatment decisions, highlighting the need for further prospective research. One of the limitations of the study is that neonatal data were not included in the analysis because only risk factors and maternal outcomes related to isthmocele were evaluated. Strengths include detailed isthmocele evaluation by an experienced physician, inclusion of both symptomatic and asymptomatic cases, and use of high-resolution ultrasound per Delphi criteria. Morphometric analysis (RMT, AMT, and ratios) enhanced structural understanding. Cesarean number (≥ 3) was an independent predictor, strengthening analytical validity.

### Conclusion and recommendations

Repeated cesarean sections are an independent risk factor for isthmocele development, with a prevalence reaching 100% in patients with four prior cesareans. Morphometric parameters, particularly reduced RMT, demonstrate a strong association with the presence of isthmocele, while a niche length ≥ 5 mm is strongly associated with symptomatology. We recommend incorporating routine morphometric ultrasound evaluation—including RMT and niche length measurements—into post-cesarean gynecological follow-up protocols, especially for patients with multiple cesarean deliveries or unexplained pelvic symptoms. This approach may facilitate earlier diagnosis and optimize management strategies. Future research should aim to establish standardized morphometric thresholds to improve clinical decision-making and treatment guidance. In addition, the development of artificial intelligence-assisted imaging tools holds promise for enhancing diagnostic accuracy, enabling more precise identification of symptomatic cases, and minimizing unnecessary interventions.

## Data Availability

I am willing to share the data I collected for the study upon request by the journal’s editorial board.
